# Prevalence and contributing factors of anemia in patients with gynecological cancer: a retrospective cohort study

**DOI:** 10.1038/s41598-024-61015-4

**Published:** 2024-05-09

**Authors:** Kexue Ning, Xingyu Sun, Ling Liu, Lijuan He

**Affiliations:** 1 College of Agroforestry and Health, The Open University of Sichuan, Chengdu, China; 2 Department of Gynecology, The Affiliated Traditional Chinese Medicine Hospital, Southwest Medical University, Luzhou, 646000 Sichuan China; 3 Department of Reproductive Medicine Center, The Affiliated Hospital, Southwest Medical University, 25 Taiping Street, Luzhou, China; 4 Department of Health Management Center, The Affiliated Hospital, Southwest Medical University, 25 Taiping Street, Luzhou, China

**Keywords:** Anemia, Gynecological Cancer, Prevalence, Risk Factors, Hemoglobin Levels, Retrospective Cohort, Public health, Quality of life

## Abstract

This retrospective cohort study aimed to determine the prevalence of anemia among patients with gynecological cancer prior to any treatment and to identify contributing factors associated with anemia in this group. We retrospectively analyzed data from female patients aged 18 and above, diagnosed with various forms of gynecological cancer at The Affiliated Hospital of Southwest Medical University between February 2016 and March 2021. Anemia was assessed based on the most recent CBC results before any cancer treatment. Eligibility was based on a definitive histopathological diagnosis. Key variables included demographic details, clinical characteristics, and blood counts, focusing on hemoglobin levels. Statistical analysis was conducted using logistic regression models, and anemia was defined as hemoglobin levels below 12 g/dL for women, according to WHO criteria. Of the 320 participants, a significant prevalence of anemia was found. Correlations between anemia and factors like age, educational level, and biological markers (iron, folic acid, and vitamin B12 levels) were identified. In our study, we found that the prevalence of anemia among patients with gynecological cancer prior to any treatment was 59.06%, indicating a significant health concern within this population. The study highlights a significant prevalence of anemia in patients with gynecological cancer, emphasizing the need for regular hemoglobin screening and individualized management. These findings suggest the importance of considering various characteristics and clinical variables in anemia management among this patient group. Further studies are needed to explore the long-term effects of these factors on patient outcomes and to develop targeted interventions.

## Introduction

Anemia, a condition characterized by a deficient number of red blood cells or low hemoglobin levels, is a global health issue affecting both developing and developed countries^1,2^. It presents a particularly concerning comorbidity in patients with various cancers, including those diagnosed with gynecological malignancies. Its prevalence in cancer patients, especially those with solid tumors, is notably high, affecting about 30% to 90%. In patients with gynecological malignancies, this prevalence ranges from 26 to 85%. The etiology of anemia in these patients is complex, involving both tumor-specific factors and treatment-related elements, such as chronic inflammation and the suppression of erythropoietin production^3^. The presence of anemia in cancer patients is associated with reduced survival, decreased quality of life, and impaired response to treatment^4–6^.

Gynecological cancers, encompassing ovarian, cervical, and endometrial cancers, represent a significant portion of cancer diagnoses in women worldwide^7^. These malignancies are often accompanied by multiple complications, with anemia being a prevalent concurrent condition, potentially due to factors such as nutritional deficiencies, chronic bleeding, iron malabsorption, or treatment-related effects^8^. Despite its prevalence, the multifactorial etiology of anemia in gynecological cancer patients remains insufficiently explored, necessitating comprehensive studies to unravel the contributing factors and impact on clinical outcomes.

Several studies have underscored the negative implications of anemia on prognosis in cancer patients. Anemic cancer patients often exhibit diminished physical function, lower overall well-being, and reduced tolerance to cancer therapies, which can compromise treatment efficacy^9^. Furthermore, anemia has been associated with poorer prognosis and decreased survival rates in various cancer types^10^. In gynecological cancers, specifically, anemia prevalence has been reported to vary, influencing treatment decisions and outcomes^11^.

Managing anemia in patients with gynecological cancer is paramount, as correction of hemoglobin levels has been shown to improve treatment response, quality of life, and survival rates^12^. However, the heterogeneity in anemia's onset, severity, and etiological factors across different gynecological cancers complicates the formulation of uniform management strategies. This complexity underscores the need for a deeper understanding of anemia's prevalence, risk factors, and impact in the context of gynecological malignancies^13^.

**Figure 1:**
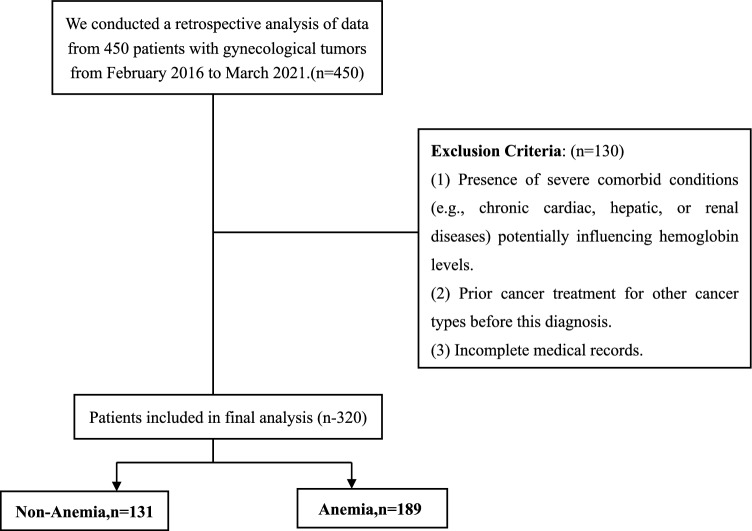
Study flowchart.

Moreover, while the global burden of anemia has been extensively studied, there are geographical and demographic disparities in the available data^10^. Most existing research focuses on populations in high-income countries, with less known about anemia's characteristics in low- and middle-income regions^14^. These gaps highlight the necessity for localized studies that consider regional medical practices, demographic factors, and access to healthcare services (Fig. 1).

This study aims to fill these gaps by exploring the prevalence and risk factors of anemia among patients with gynecological cancers in a retrospective cohort. By analyzing demographic, clinical, and laboratory data, this research seeks to identify significant predictors of anemia in this population, contributing to more personalized and effective management strategies for affected patients. The findings are expected to provide healthcare professionals with insights to enhance anemia screening, prevention, and treatment measures in patients facing gynecological cancers, ultimately aiming to improve patient quality of life and survival outcomes.

## Materials and Methods

### Study design and participants

This retrospective cohort study involved a carefully selected sample of 320 patients diagnosed with various forms of gynecological cancer, out of a larger pool of cases at The Affiliated Hospital of Southwest Medical University. The study spanned February 2016 to March 2021. Eligibility required female patients aged 18 or older with a confirmed histopathological diagnosis of gynecological cancer, including ovarian, cervical, and endometrial cancers. Comprehensive medical histories and records were essential for inclusion. Patients with severe concurrent diseases affecting hemoglobin levels, prior cancer treatments, or incomplete records were excluded. This selection ensured a focused analysis on the relationship between gynecological cancer and anemia.

### Data collection

We reviewed detailed medical records to collect demographic, clinical, and laboratory data. This included age, marital status, economic status, education level, tumor type and stage, treatment history, and more. Laboratory data focused on hemoglobin levels, red blood cell count, and other relevant parameters. All data were anonymized to uphold ethical standards.

### Outcome measures

Anemia prevalence, defined by WHO criteria (hemoglobin < 12 g/dL for women), was evaluated. Anemia status was determined from the latest CBC results before any cancer treatment, providing a baseline unaffected by treatment.

### Statistical analysis

We employed descriptive statistics, univariate analysis, and multivariate logistic regression to identify anemia predictors, using SPSS software. A p-value < 0.05 was considered statistically significant.

### Ethical considerations

The study was ethically approved, with informed consent waived due to its retrospective nature. All procedures complied with ethical standards and the Helsinki Declaration.

### Ethical approval and consent to participate

This investigation was undertaken with the sanction of the Ethics Committee of The Affiliated Hospital of Southwest Medical University (Ethics code number: KY2023200) and an exemption for informed consent was obtained from the Ethics Committee of The Affiliated Hospital of Southwest Medical University due to retrospective nature of the study. All methods were conducted in compliance with relevant guidelines, regulations, and the Declaration of Helsinki.

## Results

### Comparative analysis of demographic and clinical characteristics across overall cohort, non-anemic, and anemic patients with gynecological cancer

Table 1 presents a comprehensive comparative analysis of the demographic and clinical characteristics among the overall cohort, and distinctively between non-anemic and anemic patients diagnosed with gynecological cancer. The study encompassed a total of 320 patients, subdivided into 131 non-anemic and 189 anemic individuals based on predefined hemoglobin criteria. The median age for the entire cohort was 60 years, with a discernible age difference between non-anemic (median age 52 years) and anemic patients (median age 62 years), indicating a statistically significant association of older age with anemia (P < 0.001). Body weight and height measurements across the groups showed median values of 71 kg and 1.66 m, respectively, with no significant differences observed (Weight P = 0.492, Height P = 0.805). Similarly, Body Mass Index (BMI) comparisons revealed a median of 26.139 for the overall cohort, showing no significant difference between the non-anemic and anemic groups (P = 0.634). The distribution of marital status, economic status, and education level across the study population demonstrated a varied demographic profile with no significant differences in these socio-economic factors between non-anemic and anemic patients. This is highlighted by the comparable percentages across marital statuses and the slight variances in economic status and education levels that did not reach statistical significance. Clinically, hemoglobin levels displayed a marked difference, serving as the basis for distinguishing between anemic and non-anemic participants. The mean hemoglobin concentration was significantly lower in anemic patients (11.2 g/dL) compared to non-anemic patients (13.3 g/dL, P < 0.001). The analysis further explored red blood cell count, hematocrit, and mean cell volume, with no significant differences found between the two groups, emphasizing the specific impact of hemoglobin levels on anemia classification in this context. A closer look at biochemical markers revealed statistically significant lower levels of iron, folic acid, and vitamin B12 in anemic patients compared to non-anemic ones, underscoring the nutritional and metabolic factors contributing to anemia in this patient population. The types of tumors also showed a significant association with anemia prevalence, particularly noting a higher occurrence of cervical cancer among anemic patients, while the distribution of tumor stages and treatment history across both groups showed no statistical significance, indicating the inherent nature of anemia as a condition influencing the patient group regardless of cancer stage or treatment modality. In terms of reproductive health history, menstrual regularity and childbirth counts were considered, revealing no significant differences between the anemic and non-anemic groups, thus indicating the multifactorial causes of anemia beyond reproductive factors. The assessment of complications, medication history, nutrition intake, quality of life, and prognosis did not exhibit significant differences between the two groups, further emphasizing the complex interplay of factors contributing to anemia in patients with gynecological cancer.

**Table 1:** Comparative Analysis of Demographic and Clinical Characteristics Across Overall Cohort, Non-Anemic, and Anemic Patients with Gynecological Cancer.

Characteristics	overall	Non-Anemia	Anemia	P value
n	320	131	189	
Age, median (IQR)	60 (45.75, 72)	52 (43.5, 69.5)	62 (48, 73)	< 0.001
Weight, median (IQR)	71 (55, 86.25)	71 (57.5, 86)	71 (54, 87)	0.492
Height, median (IQR)	1.66 (1.59, 1.73)	1.67 (1.59, 1.73)	1.66 (1.57, 1.73)	0.805
BMI, median (IQR)	26.139 (19.84, 31.104)	25.881 (21.064, 30.965)	26.396 (19.396, 31.121)	0.634
Marital Status, n (%)				0.874
Divorced	82 (25.6%)	32 (10%)	50 (15.6%)	
Widowed	81 (25.3%)	34 (10.6%)	47 (14.7%)	
Single	73 (22.8%)	28 (8.8%)	45 (14.1%)	
Married	84 (26.2%)	37 (11.6%)	47 (14.7%)	
Economic Status, n (%)				0.900
High	109 (34.1%)	43 (13.4%)	66 (20.6%)	
Middle	110 (34.4%)	45 (14.1%)	65 (20.3%)	
Low	101 (31.6%)	43 (13.4%)	58 (18.1%)	
Education Level, n (%)				0.135
Secondary	83 (25.9%)	40 (12.5%)	43 (13.4%)	
Primary	72 (22.5%)	24 (7.5%)	48 (15%)	
Tertiary	85 (26.6%)	39 (12.2%)	46 (14.4%)	
Postgraduate	80 (25%)	28 (8.8%)	52 (16.2%)	
Family History of Gynecological Tumor or Anemia, n (%)				0.605
Yes	157 (49.1%)	62 (19.4%)	95 (29.7%)	
No	163 (50.9%)	69 (21.6%)	94 (29.4%)	
Hemoglobin Levels, median (IQR)	11.963 ± 1.4685	13.3 (12.65, 13.9)	11.2 (10.4, 11.6)	< 0.001
Red Blood Cell Count, mean ± sd	5.08 ± 0.82274	5.1649 ± 0.8045	5.0212 ± 0.83218	0.125
Hematocrit, median (IQR)	42.5 (39.5, 46.425)	43.1 (40.05, 46.45)	41.9 (39.3, 46.4)	0.104
Mean Cell Volume, mean ± sd	90 ± 6.8503	89.702 ± 6.998	90.206 ± 6.757	0.518
Iron Levels, mean ± sd	52.776 ± 19.563	57.8 ± 19.74	49.294 ± 18.712	< 0.001
Folic Acid Levels, mean ± sd	8.8477 ± 3.1146	10.124 ± 2.9921	7.9628 ± 2.8889	< 0.001
Vitamin B12 Levels, mean ± sd	446.99 ± 165.96	504.38 ± 163.08	407.21 ± 156.4	< 0.001
Bone Marrow Result, n (%)				0.271
Normal	173 (54.1%)	66 (20.6%)	107 (33.4%)	
Abnormal	147 (45.9%)	65 (20.3%)	82 (25.6%)	
Tumor Type, n (%)				< 0.001
Ovarian	106 (33.1%)	46 (14.4%)	60 (18.8%)	
Cervical	112 (35%)	29 (9.1%)	83 (25.9%)	
Endometrial	102 (31.9%)	56 (17.5%)	46 (14.4%)	
Tumor Stage, n (%)				0.550
III	77 (24.1%)	30 (9.4%)	47 (14.7%)	
IV	75 (23.4%)	36 (11.2%)	39 (12.2%)	
I	98 (30.6%)	37 (11.6%)	61 (19.1%)	
II	70 (21.9%)	28 (8.8%)	42 (13.1%)	
Treatment History, n (%)				0.846
Chemotherapy	70 (21.9%)	29 (9.1%)	41 (12.8%)	
Surgery	83 (25.9%)	36 (11.2%)	47 (14.7%)	
Targeted_Therapy	73 (22.8%)	31 (9.7%)	42 (13.1%)	
Radiotherapy	94 (29.4%)	35 (10.9%)	59 (18.4%)	
Menstrual History, n (%)				0.107
Irregular	159 (49.7%)	58 (18.1%)	101 (31.6%)	
Regular	161 (50.3%)	73 (22.8%)	88 (27.5%)	
Childbirth Count, median (IQR)	3 (1, 4)	2 (1, 4)	3 (1, 4)	0.242
Complications, n (%)				0.939
Infection	81 (25.3%)	35 (10.9%)	46 (14.4%)	
None	89 (27.8%)	36 (11.2%)	53 (16.6%)	
Bleeding	93 (29.1%)	36 (11.2%)	57 (17.8%)	
Thrombosis	57 (17.8%)	24 (7.5%)	33 (10.3%)	
Medication History, n (%)				0.140
Vitamin B12 Supplements	73 (22.8%)	22 (6.9%)	51 (15.9%)	
Folic Acid Supplements	86 (26.9%)	40 (12.5%)	46 (14.4%)	
Iron Supplements	76 (23.8%)	35 (10.9%)	41 (12.8%)	
None	85 (26.6%)	34 (10.6%)	51 (15.9%)	
Nutrition Intake, n (%)				0.749
Inadequate	170 (53.1%)	71 (22.2%)	99 (30.9%)	
Adequate	150 (46.9%)	60 (18.8%)	90 (28.1%)	
Quality of Life, n (%)				0.552
Poor	96 (30%)	36 (11.2%)	60 (18.8%)	
Good	114 (35.6%)	51 (15.9%)	63 (19.7%)	
Average	110 (34.4%)	44 (13.8%)	66 (20.6%)	
Prognosis, n (%)				0.829
Survived	105 (32.8%)	43 (13.4%)	62 (19.4%)	
Deceased	108 (33.8%)	42 (13.1%)	66 (20.6%)	
Relapsed	107 (33.4%)	46 (14.4%)	61 (19.1%)	

This table presents a comprehensive comparison of demographic, clinical, and laboratory characteristics across the entire study cohort (n = 320), further delineated between non-anemic (n = 131) and anemic (n = 189) patients. Values for continuous variables are expressed as median (Interquartile Range, IQR) for non-normally distributed data, and mean ± standard deviation (SD) for normally distributed data. Categorical variables are represented as counts (n) and percentages (%). Statistical significance between non-anemic and anemic groups was assessed using the Mann–Whitney U test for continuous variables and the Chi-square or Fisher's exact test for categorical variables, with a P value of < 0.05 indicating statistical significance. This combined analysis aims to provide an in-depth understanding of the demographic and clinical landscape of our study population, emphasizing the distinct characteristics associated with anemia status.

### Univariate and multivariate analyses of factors associated with anemia in patients with gynecological cancer

Table 2 presents a comprehensive analysis of the various factors potentially influencing the prevalence of anemia among the studied population. The multivariate analysis, which adjusts for potentially confounding variables identified in the univariate analysis, highlights several parameters with statistically significant associations with anemia. Age demonstrated a notable influence, with an odds ratio of 1.034, indicating that as the participants' age increased, so did the likelihood of anemia, a relationship that was statistically significant (P < 0.001). This finding underscores the importance of age as a factor in anemia prevalence. Interestingly, educational level emerged as another significant factor. Individuals with primary education levels were significantly more likely to experience anemia, with an odds ratio of 2.479 (P = 0.026), compared to those with secondary education levels. Furthermore, postgraduates showed an increased likelihood of anemia, with an odds ratio of 2.235, which was also statistically significant (P = 0.039). Several biological markers were prominently associated with anemia. Lower iron levels, lower folic acid levels, and lower vitamin B12 levels were all significantly associated with a higher likelihood of anemia, with P values of < 0.001, indicating strong statistical significance. These findings reinforce the known biological pathways of anemia, where deficiencies in these critical components often manifest in anemic symptoms. In terms of gynecological health, the type of tumor also influenced anemia prevalence. Specifically, individuals with cervical tumors were more likely to be anemic, with an odds ratio of 1.933, though this result bordered on statistical significance (P = 0.056). In contrast, several factors, including marital status, economic status, family history, and certain health markers (red blood cell count, hematocrit, mean cell volume), did not exhibit a significant association with anemia, underscoring the complexity of anemia's etiology. Overall, Table 2 elucidates the multifaceted nature of anemia's contributing factors, emphasizing the need for a holistic approach to patient assessment and treatment. By understanding these associations, healthcare professionals can better identify at-risk individuals and implement appropriate preventive and therapeutic measures.

**Table 2:** Univariate and Multivariate Analyses of Factors Associated with Anemia in Patients with Gynecological Cancer.

Characteristics	Total(N)	Univariate analysis		Multivariate analysis	
		Odds Ratio (95% CI)	P value	Odds Ratio (95% CI)	P value
Age	320	1.030 (1.015—1.046)	** < 0.001**	1.034 (1.015—1.053)	** < 0.001**
Marital Status	320				
Divorced	82	Reference			
Widowed	81	0.885 (0.473—1.654)	0.701		
Single	73	1.029 (0.538—1.966)	0.932		
Married	84	0.813 (0.438—1.509)	0.512		
Economic Status	320				
High	109	Reference			
Middle	110	0.941 (0.548—1.616)	0.826		
Low	101	0.879 (0.507—1.524)	0.646		
Education Level	320				
Secondary	83	Reference		Reference	
Primary	72	1.860 (0.969—3.572)	0.062	2.479 (1.115—5.513)	**0.026**
Tertiary	85	1.097 (0.598—2.011)	0.764	1.294 (0.613—2.731)	0.498
Postgraduate	80	1.728 (0.920—3.243)	0.089	2.235 (1.040—4.802)	**0.039**
Family History of Gynecological Tumor or Anemia	320				
Yes	157	Reference			
No	163	0.889 (0.569—1.389)	0.605		
Red Blood Cell Count	320	0.807 (0.614—1.061)	0.125		
Hematocrit	320	0.957 (0.905—1.011)	0.117		
Mean Cell Volume	320	1.011 (0.978—1.044)	0.516		
Iron Levels	320	0.977 (0.965—0.989)	** < 0.001**	0.977 (0.963—0.991)	**0.001**
Folic Acid Levels	320	0.774 (0.710—0.844)	** < 0.001**	0.770 (0.697—0.851)	** < 0.001**
Vitamin B12 Levels	320	0.996 (0.995—0.998)	** < 0.001**	0.996 (0.994—0.998)	** < 0.001**
Bone Marrow Result	320				
Normal	173	Reference			
Abnormal	147	0.778 (0.498—1.217)	0.272		
Tumor Type	320				
Ovarian	106	Reference		Reference	
Cervical	112	2.194 (1.239—3.885)	**0.007**	1.933 (0.983—3.801)	0.056
Endometrial	102	0.630 (0.364—1.089)	0.098	0.628 (0.324—1.217)	0.168
Tumor Stage	320				
III	77	Reference			
IV	75	0.691 (0.363—1.317)	0.262		
I	98	1.052 (0.570—1.944)	0.871		
II	70	0.957 (0.494—1.856)	0.898		
Treatment History	320				
Chemotherapy	70	Reference			
Surgery	83	0.923 (0.485—1.758)	0.808		
Targeted_Therapy	73	0.958 (0.493—1.862)	0.900		
Radiotherapy	94	1.192 (0.633—2.246)	0.586		
Menstrual History	320				
Irregular	159	Reference			
Regular	161	0.692 (0.442—1.083)	0.107		
Childbirth Count	320	1.080 (0.941—1.239)	0.272		
Complications	320				
Infection	81	Reference			
None	89	1.120 (0.608—2.062)	0.716		
Bleeding	93	1.205 (0.657—2.209)	0.547		
Thrombosis	57	1.046 (0.527—2.076)	0.897		
Medication History	320				
Vitamin B12 Supplements	73	Reference		Reference	
Folic Acid Supplements	86	0.496 (0.258—0.955)	**0.036**	0.518 (0.267—1.005)	0.052
Iron Supplements	76	0.505 (0.258—0.991)	**0.047**	0.515 (0.261—1.014)	0.055
None	85	0.647 (0.334—1.254)	0.197	0.654 (0.336—1.274)	0.212
Nutrition Intake	320				
Inadequate	170	Reference			
Adequate	150	1.076 (0.688—1.682)	0.749		
Quality of Life	320				
Poor	96	Reference			
Good	114	0.741 (0.426—1.290)	0.289		
Average	110	0.900 (0.513—1.579)	0.713		
Prognosis	320				
Survived	105	Reference			
Deceased	108	1.090 (0.630—1.886)	0.759		
Relapsed	107	0.920 (0.533—1.587)	0.764		

This table presents the results of univariate and multivariate logistic regression analyses assessing various factors associated with the risk of anemia. The Odds Ratios (ORs) and 95% Confidence Intervals (CIs) provide estimates of the effect size of each factor on the risk of anemia.

A P-value of < 0.05 was considered statistically significant. In the multivariate analysis, adjustments were made for all variables that showed potential relevance in the univariate analysis. Significant values are in bold.

## Discussion

This study illuminated several critical factors associated with anemia among patients with gynecological cancer, drawing attention to the intricate interplay between demographic, clinical, and socioeconomic variables. The findings underscore the necessity for a multifaceted approach to patient care, considering not only clinical symptoms but also the broader social determinants of health.

Age emerged as a significant predictor of anemia, with older patients exhibiting a higher likelihood of this condition. This trend aligns with existing research that has documented physiological changes related to aging, such as decreased bone marrow response and nutritional deficiencies, contributing to anemia's development^15,16^. Furthermore, older individuals often have comorbid conditions, complicating their clinical presentations^17^. Our study reinforces the importance of comprehensive geriatric assessments and tailored care strategies, acknowledging the unique physiological and social challenges this demographic faces.

The association between anemia and specific gynecological cancers, particularly cervical cancer, was a notable discovery. This outcome suggests that the biological characteristics of tumors, possibly related to their metabolic demands or cytokine-mediated systemic effects, play a role in modulating anemia risk^18–20^. These findings underscore the necessity for tumor-specific screening protocols and possibly differential management strategies, catering to the individualized needs of patients based on their cancer type.

Our study's revelation of the strong association between anemia and deficiencies in iron, folic acid, and vitamin B12 amplifies the conversation around holistic patient care. It's a reminder that clinical management should extend beyond treating cancer itself, encompassing aspects like nutritional counseling^21^. These deficiencies could be reflective of broader issues, including dietary habits, socioeconomic status, and even the impact of cancer therapies^22^. Incorporating nutritional assessments and interventions into patient care protocols could mitigate these risk factors, potentially improving treatment outcomes and quality of life.

The socioeconomic and educational disparities highlighted in our findings present a more systemic challenge. Lower educational levels correlated with higher anemia prevalence, potentially indicating gaps in health literacy, accessibility to healthcare resources, and overall health awareness^23^. This observation aligns with existing literature documenting health outcome disparities based on socioeconomic status^24^. It's a call to action for healthcare systems to adopt more inclusive strategies, ensuring that education, economic background, or social circumstances do not disadvantage patients in their healthcare journeys.

The insights gleaned from this study have several implications for clinical practice. They advocate for a more integrated approach to care, encompassing routine anemia screening, nutritional counseling, and targeted interventions for at-risk demographics. Furthermore, healthcare providers should be cognizant of the broader socioeconomic factors at play, advocating where possible for policy changes or support mechanisms to bridge these gaps.

It is crucial to acknowledge the role of our study's exclusion criteria, particularly the decision to omit patients with severe concurrent diseases known to affect hemoglobin levels. This choice was aimed at minimizing confounding factors and isolating the impact of gynecological cancers on anemia. However, this approach also means that the broader influence of comorbidities, such as chronic kidney disease, inflammatory diseases, and nutritional deficiencies, was not directly addressed within our analysis.

In reflecting upon the scope of our study, it is essential to acknowledge certain limitations that bear implications for the interpretation of our findings. The retrospective nature of our analysis, while offering a comprehensive overview, inherently limits our ability to infer causality between the incidence of anemia and gynecological cancers. Moreover, our examination did not extend into depth regarding lifestyle factors and other possible contributors to anemia, potentially overlooking significant determinants of its prevalence. A particularly noteworthy consideration is the variance in anemia prevalence across different stages of cervical cancer, which may be attributed to factors such as bleeding in advanced stages. This aspect was not delineated in our study, suggesting a pivotal area for subsequent research to explore the impact of cancer progression on anemia. Additionally, the homogeneity of our study population restricts the extrapolation of our findings to more diverse demographic groups. This limitation underscores the need for future research endeavors to embrace a broader demographic spectrum, thereby enhancing the generalizability and applicability of the findings. These considerations highlight the necessity for future studies to adopt a prospective design for establishing causality, delve deeper into the multifaceted contributors to anemia, and assess the influence of cancer staging on its prevalence, especially in the context of cervical cancer. By addressing these gaps, the research community can further enrich our understanding and management strategies for anemia in patients with gynecological cancer.

In addressing the crucial aspect of anemia prevalence among patients with gynecological cancers before the commencement of any treatment, our findings reveal a significant rate of 59.06%. This rate is particularly noteworthy in the context of the broader literature on the subject. For example, research conducted by Alghamdi et al. (2021) at King Abdulaziz Medical City, Jeddah, identified a prevalence rate of 90.7% among patients receiving active treatment, highlighting the impact of chemotherapy and radiotherapy on hemoglobin levels ^11^. The difference between these rates underscores the importance of recognizing anemia as a pre-existing condition in a considerable proportion of gynecological cancer patients, which may be further exacerbated by the treatment process.

The distinction between pre-treatment and treatment-induced anemia emphasizes the necessity for early detection and management strategies tailored to address this condition from the point of cancer diagnosis. Integrating anemia management into the overall treatment plan for gynecological cancers is crucial, not only to improve patient quality of life but also potentially to enhance the efficacy of cancer treatment protocols.

Our study contributes to the growing body of evidence suggesting that anemia is a multifactorial issue in the context of gynecological cancers, with implications for both pre-treatment condition management and the monitoring of treatment-related side effects. It highlights the need for a proactive approach to anemia screening and intervention, ensuring comprehensive patient care that addresses all facets of this condition.

In conclusion, this study underscores the multifactorial nature of anemia in patients with gynecological cancer, highlighting the influence of demographic, tumor-specific, nutritional, and socioeconomic factors. The findings advocate for an integrated, patient-centered approach to care, sensitive to the various challenges patients may face in their healthcare journeys. As we move forward, a commitment to continual research and an embrace of holistic care strategies will be paramount in enhancing patient outcomes and quality of life.

## Dara availability

The datasets analyzed during the current study are not publicly available due to privacy but are available from the corresponding author at a reasonable request.
